# Tubular filamentation for laser material processing

**DOI:** 10.1038/srep08914

**Published:** 2015-03-10

**Authors:** Chen Xie, Vytautas Jukna, Carles Milián, Remo Giust, Ismail Ouadghiri-Idrissi, Tatiana Itina, John M. Dudley, Arnaud Couairon, Francois Courvoisier

**Affiliations:** 1 Département d'Optique P. M. Duffieux, Institut FEMTO-ST, UMR 6174 CNRS Université de Franche-Comté, F-25030 Besançon cedex, France; 2 Centre de Physique Théorique, CNRS, Ecole Polytechnique, F-91128 Palaiseau, France; 3 Laboratoire Hubert Curien, UMR CNRS 5516, Université de Lyon, Université Jean Monnet, F-42000 Saint-Etienne, France

## Abstract

An open challenge in the important field of femtosecond laser material processing is the controlled internal structuring of dielectric materials. Although the availability of high energy high repetition rate femtosecond lasers has led to many advances in this field, writing structures within transparent dielectrics at intensities exceeding 10^13^ W/cm^2^ has remained difficult as it is associated with significant nonlinear spatial distortion. This letter reports the existence of a new propagation regime for femtosecond pulses at high power that overcomes this challenge, associated with the generation of a hollow uniform and intense light tube that remains propagation invariant even at intensities associated with dense plasma formation. This regime is seeded from higher order nondiffracting Bessel beams, which carry an optical vortex charge. Numerical simulations are quantitatively confirmed by experiments where a novel experimental approach allows direct imaging of the 3D fluence distribution within transparent solids. We also analyze the transitions to other propagation regimes in near and far fields. We demonstrate how the generation of plasma in this tubular geometry can lead to applications in ultrafast laser material processing in terms of single shot index writing, and discuss how it opens important perspectives for material compression and filamentation guiding in atmosphere.

In 1992, Allen et al demonstrated that light beams with a phase singularity or vortex charge (i.e. orbital angular momentum) can transfer mechanical torque^1^. These waves attracted intense interest for optical manipulation of micro and nano-objects^2^. The vortex singularity was identified as a supplementary degree of freedom for optical encoding and applications emerged in a diversity of fields such as optical metrology^3^, nonlinear optics^4^,^5^,^6^, and quantum information^7^,^8^.

In this paper, we report on an important novel application of vortex beams to control nonlinear propagation of intense femtosecond light beams within dielectrics, and demonstrate its relevance for laser material processing. In particular, for the first time to our knowledge, ultra-intense light pulses are shown to be able to propagate as a “light tube” in dielectrics without deformation, generating a tubular plasma distribution that reaches optical breakdown densities. This novel tubular geometry for delivery of energy from femtosecond pulses in a propagation-invariant way is expected to generate novel breakthroughs in femtosecond laser material processing.

The complex nonlinear dynamics of femtosecond laser pulse propagation in dielectrics generally inhibits uniform energy deposition within the material along the beam path. The addition of a vortex singularity on a Gaussian beam in the form of a doughnut has been shown to introduce some additional propagation control^9^, associated with azimuthal modulation instability, filament formation and soliton dynamics^10^,^11^,^12^. Several solitonic regimes have been identified, where the beam can either remain doughnut shaped or split into several rotating filaments^9^. However, this solitonic behavior which would be essential for material processing stops at higher intensity where strong nonlinear losses occur.

In contrast to Gaussian beams, however, nondiffracting Bessel beams have been successfully shown to sustain invariant propagation even in presence of nonlinear losses and Kerr effect^13^. This arises physically because of the particular property of Bessel beams where conical inward energy flow can compensate for losses and “self-heal” the beam during propagation^14^. Experimental results have applied this propagation-invariance of zero-order femtosecond Bessel beams to single shot laser processing of nanochannels in glass with high aspect ratios from 100:1 to 1000:1^15^,^16^.

In this paper, we investigate nonlinear femtosecond Bessel beams carrying a vortex charge. The concept is shown in Figure 1. The beam structure consists of a primary “light tube” of high intensity surrounded by several other concentric tubes at lower intensity. This is shown in Figure 1(a). With linear propagation, the intensity distribution is quasi-invariant along z^17^. The radial profile of Bessel beams carrying a vortex charge *m* is well approximated by the Bessel function of order *m*: *I*(*r*) ~ |*J_m_*(*k*sin*γ r*)|^2^ where *k* is the wavevector and *γ* is the conical angle. As will be discussed below, the conical angle is a parameter of primary importance. For *m* = 0, the tube radius is null and the conical angle is the angle that geometrical rays make with the optical axis. For *m* ≥ 1, geometrical analysis shows that light propagates tangentially to the tube and no light crosses the inner part of the tubular main intensity region^18^. The experimental setup used to generate the beams and image their propagation is shown in Figure 1(b) and this is discussed in more detail later.

This paper is organized as follows. We first describe propagation-invariant vortex solutions to the nonlinear Schrödinger propagation equation, including the Kerr effect and nonlinear losses. We then analyze with numerical simulations the domain of existence of such solutions that we refer to as *propagation-invariant conical vortex waves*, and we see how nonlinear propagation is attracted toward these propagation-invariant solutions. To experimentally confirm our numerical results, we needed an accurate technique to record beam propagation. Spatiotemporal measurements of nonlinear propagation have already been developed within liquids by using a cuvette with variable thickness^19^. Here, for propagation within solids, we have developed a novel experimental approach. Based on beam scanning, our setup allows for a direct imaging of the 3D fluence distribution within transparent solids in near and far field. *Quantitative* agreement is found with numerical simulations. We discuss our results in terms of three broad regimes of propagation: (i) *tubular propagation-invariant* where the intensity distribution pattern does not vary with the propagation distance, (ii) *rotating*, where several filaments rotate around the optical axis during the propagation and (iii) *speckle-like*, where hot-spots are non-rotating, and appear and disappear in space.

## Results

### Propagation-invariant solutions to nonlinear propagation in dielectrics

The key physical effects in the physics of stationary conical vortex waves are diffraction, the optical Kerr effect and nonlinear losses. The nonlinear Schrödinger equation (NLSE) describing the propagation of a field envelope *E* in dielectrics is:


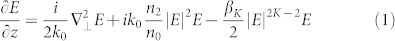
where *k*_0_ is the wavevector, 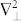 is the transverse Laplacian operator, *n*_0_ and *n*_2_ are respectively the refractive and Kerr indices of the medium, and *β_K_* is the cross section for multiphoton absorption.

We seek propagation-invariant monochromatic solutions in the form of a vortex of charge *m*, such that the electric field amplitude is: *E*(*r, ϑ, z*) = *a*(*r*)exp(−*ik_z_z* + *iϕ*(*r*) + *imϑ*). *a*(*r*) and *ϕ*(*r*) are to be determined while the longitudinal component of the wavevector, *k_z_* = *k*_0_cos*γ*, is a parameter that can be arbitrarily chosen. Our mathematical procedure is summarized in the methods section and more details are provided in Ref. 20. It follows a similar approach as in references 13, 21. In the linear regime (*n*_2_ = 0, *β_K_* = 0), equation (1) admits stationary solutions in the form of Bessel functions *a*(*r*) = *J_m_*(*k*_0_sin*γ r*), also known as “diffraction-free” solutions. In the nonlinear regime, we fixed the material parameters *n*_2_ and *β_K_* and we used numerical integration to find *a*(*r*) and *ϕ*(*r*). For a fixed *k_z_*, or equivalently a fixed conical angle, we find a family of solutions characterized by different maximum intensities. Figure 2(a) compares the radial intensity profiles of the stationary solutions found with vortex charge m = 3 and conical angle *γ* = 6.8° in fused silica, for peak intensities from 10^13^ W.cm^−2^ to 10^14^ W.cm^−2^. It is apparent that the nonlinear solutions exhibit transverse profile very similar to the linear profile except that the intensity rings are compressed and with attenuated contrast depending on material parameters and effective nonlinear losses. Contrast attenuation usually occurs for solutions reaching the highest peak intensity.

We found numerically that there is a limit in maximal intensity for each cone angle and vortex charge, over which no propagation-invariant solution is found. In Figure 2(b), we show in white (resp. white and light grey) the domain of existence of propagation invariant solutions in the parameter space determined by peak intensity and conical angle for a vortex charge m = 3 (resp. m = 1) in fused silica. The location of the frontier (red for m = 3 and blue for m = 1) obviously depends on material parameters and vortex order but remains approximately linear with positive slope. The error bars shown correspond to the accuracy to which numerical integration could locate the frontiers.

This shows that increasing cone angle allows stationary solutions to exist in a broader range of intensities where propagation-invariant solutions can be found. More importantly, this result is qualitatively valid for any kind of nonlinear losses, including those occurring by plasma absorption. This is a major result for the effectiveness of propagation-invariant conical vortex waves to applications requiring high peak powers such as femtosecond laser micromachining.

As with Bessel beams, ideal propagation-invariant conical vortex waves are only weakly localized, i.e., their amplitude tails decay as 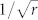 and carry infinite power. As will be shown below, a finite power beam carrying a suitably designed spatial phase will reshape into apodized versions of propagation-invariant conical vortex waves.

### Finite energy solutions and experimental results

Here, we compare experimental and numerical propagation of finite energy nonlinear Bessel vortices with the propagation-invariant solutions found above.

#### Numerical model

We produce higher order Bessel beams from a Gaussian beam by using a phase mask Φ(*r,θ*) = *k*sin*γ r* + *mθ*^17^. This is equivalent to the phase applied by an axicon and a vortex phase plate of order *m*. The numerical propagation model is detailed in the methods section. Briefly, it takes the canonical form of a unidirectional envelope propagation equation (UPPE) written in the spectral domain^22^, coupled to a plasma equation including photoionization, avalanche and recombination (see methods section). In our conditions, temporal variations are negligible and for computational efficiency in 3D, we consider the electric field *E*(*x, y, z*) as independent of time.

#### Experimental setup

**Figure 1:**
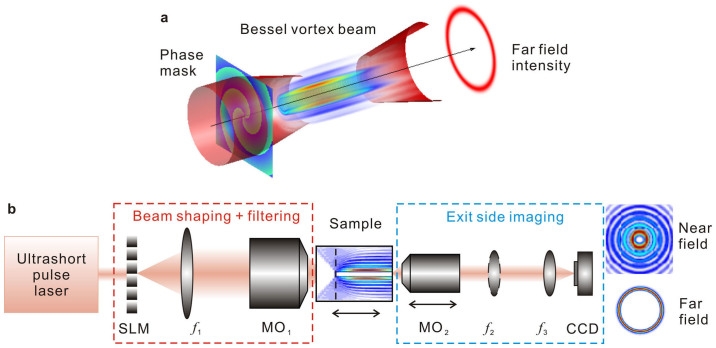
Experimental concept. (a) Conical structure of Bessel vortex beams (b) Experimental setup. (SLM) Spatial Light Modulator. (L_1_, L_2_, L_3_) are convex lenses. MO: Microscope objective. The dashed vertical line in the sample shows the position of the image of the SLM within the sample. Its relative position in the sample can be varied by translating the sample. MO_2_ is translated in equal amount to image the sample exit side. Lens L_2_ is inserted to record the far field.

Our experimental setup is shown in figure 1(b). The vortex Bessel beam is generated from the image of the SLM phase mask, placed at the focal plane of a high numerical aperture microscope objective. This allows us to generate the beam within the bulk of solid dielectrics. We have developed a novel beam procedure to image the fluence distribution in 3D for quantitative comparison with simulations. It is based on progressive precise scanning of the beam within the sample and single shot imaging of the sample exit side. The procedure is detailed in the methods section.

#### Quasi-invariant propagation of finite energy beams

A first set of experiments and numerical simulations investigated propagation of high conical angle Bessel vortices, with *γ* = 6.8° in glass. Figure 3 shows the results for several input pulse energies. The subfigures in the first and second rows show the numerical and experimental longitudinal fluence distributions. The third row compares the transverse cross-sections for a propagation distance of 300 μm from the phase mask image in the sample. Aside from the high degree of agreement between simulation and experiment, it is apparent that both numerically and experimentally, no distortion of the central main ring appears even at high input energies: only the contrast between the main ring and the surrounding rings decreases. The transverse profile changes only smoothly along the propagation, which shows the propagation is quasi-propagation-invariant.

Experimentally recorded fluences exceed 3 J/cm^2^, above material damage threshold of dielectrics. (We note that material removal or material modification occurs at a timescale much larger than the light propagation scale so that it does not affect the intensity distribution^23^).

**Figure 2:**
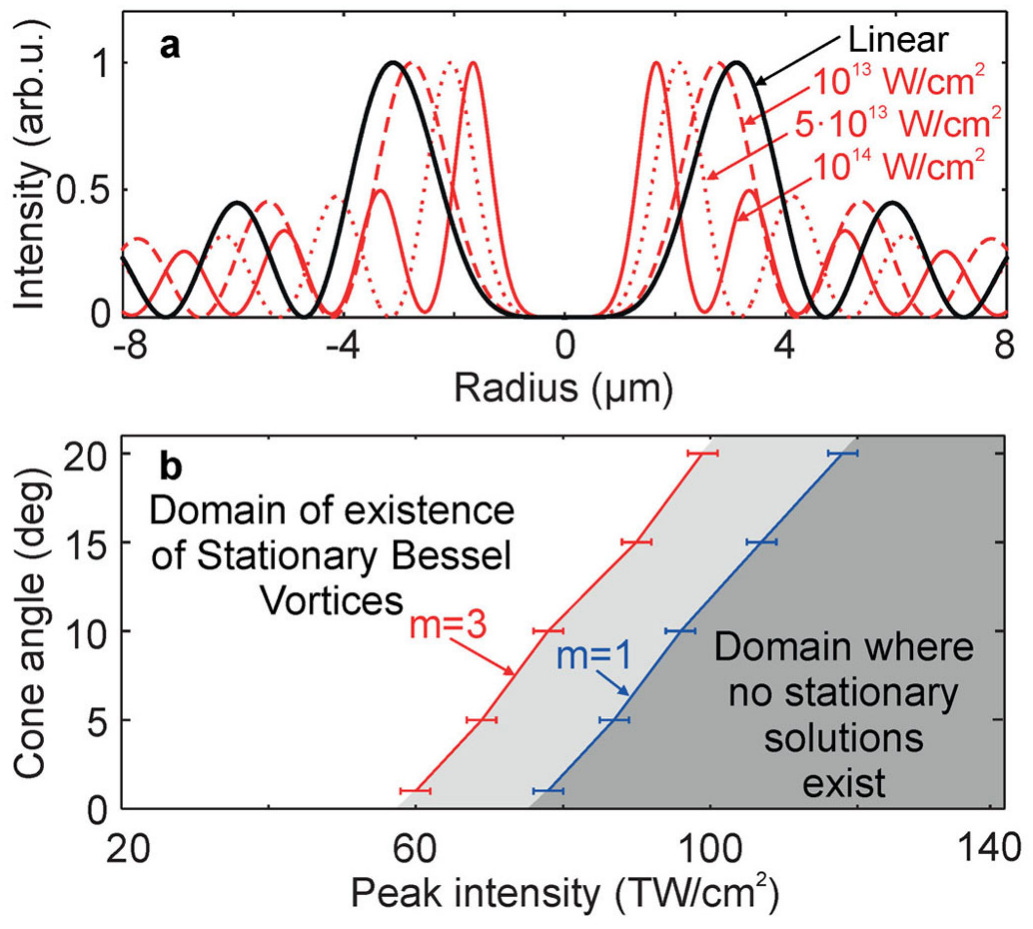
Monochromatic propagation-invariant solutions. (a) Transverse intensity profile of 4 stationary solutions corresponding to the same conical angle 6.8°, with peak intensities of 10 (dashed red), 50 (dotted red), 100 TW.cm^−2^ (solid red) and linear regime (solid black line). (b) Domains of existence and absence of stationary solutions to equation (1), in the parameter space cone angle versus peak intensity. The frontiers between the two domains for vortex charge m = 3 and m = 1 are respectively shown in red and blue. Stationary Bessel vortices exist for m = 3 (resp. m = 1) in the region shown in white (resp. white and light grey regions).

**Figure 3:**
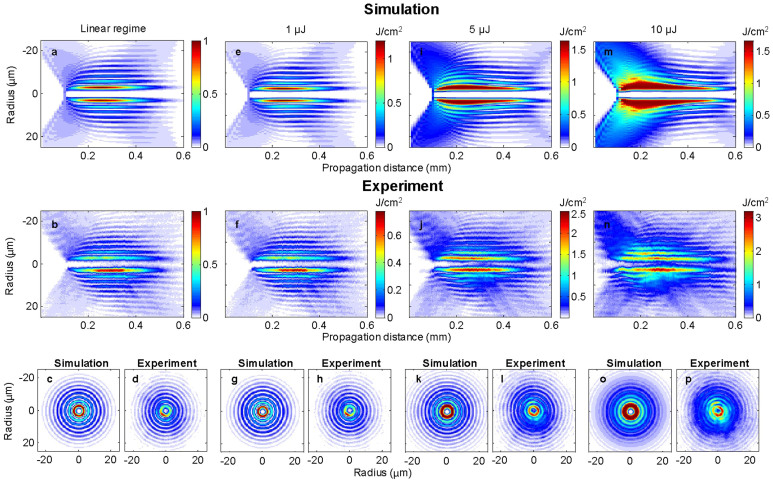
Stationary regime of Nonlinear Bessel Vortices. The fluence distribution, in J.cm^−2^, is recorded for different input pulse energies. The cone angle is 6.8°, vortex charge m = 3, and pulse duration is 120 fs. First two rows show the longitudinal fluence distribution. (top row) Numerical simulations (central row) Experimental results. (Bottom row) Comparisons of simulations and experiments for the transverse cross sections at z = 0.3 mm where the highest intensity is reached in the linear regime.

We have numerically analyzed the quasi-propagation-invariant regime in terms of peak intensity and radial position of the main intensity ring for each propagation distance. We find that the actual propagation of finite energy pulses follows a *family* of propagation-invariant conical vortex waves, such as those shown in figure 2. This property is general for conical waves^24^. This family is characterized by the *same conical angle as the one of the input beam*. In other terms, high intensity Bessel vortices reshape into a set of propagation-invariant solutions during the nonlinear propagation. Importantly, unlike solitons, this propagation-invariant solution sustains high losses occurring in the main ring. This is because a power flux from the external rings toward the main ring compensates the losses. For instance, energy loss at pulse energy of 5 μJ in Figure 3 exceeds 20% (see Methods section).

We observed both numerically and experimentally that the deviation from a quasi-propagation-invariant regime occurs because of the appearance of nonlinear wave mixing, which generates novel spatial frequencies in the beam. As observed in the case of zeroth order Bessel beams^14^,^25^, the growth of modulation instability or four-wave mixing is inefficient at high conical angle, such as for the results shown in figure 3. The value of the conical angle *γ* = 6.8° was chosen to observe a clear quasi-stationary propagation regime at high pulse energy over a long distance. To observe the departure from propagation-invariant regime, we investigate the nonlinear propagation at lower cone angle, where nonlinear wave mixing growth is reached well before dense plasma generation limits the peak intensity.

#### Transitions from propagation-invariant to rotating and speckle-like regimes

**Figure 4:**
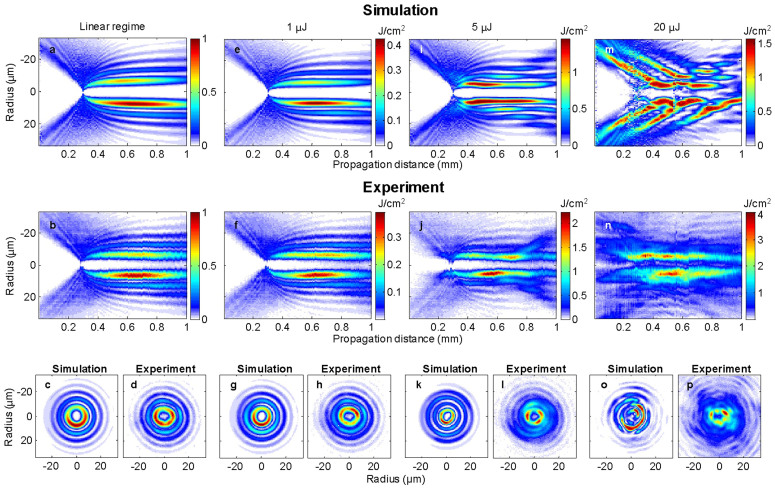
Linear, stationary, rotating and “speckle-like” regimes. They are reached during the propagation for low conical angle (2.8°) and vortex charge m = 3, when energy is progressively increased. (Top row) Numerical simulations starting from slightly distorted beam transverse profile. (Central row) Experimental results. (Bottom row) Comparison of the transverse numerical and experimental fluence profiles at a propagation distance z = 0.82 mm, where each regime is fully developed.

Figure 4 compares numerically and experimentally the fluence distribution with increasing pulse energies for a vortex of order m = 3 and *γ* = 2.8° in fused silica. The role of initial noise or beam inhomogeneity is essential to seed modulation instability and four wave mixing. We reproduced numerically the beam inhomogeneity in the amplitude profile of the input experimental beam by taking into account a slight astigmatism from our laser source, where the horizontal and vertical waists of the input Gaussian beam differ by a factor 1.05. With this, we obtained an excellent quantitative agreement with our experimental data. The remaining discrepancies are attributed to the use of frozen-time simulations (see Methods section) and to incomplete plasma/light interaction model. In the bottom row, we compare numerical and experimental cross sections at the propagation distance z = 0.82 mm, where the new regimes are developed. In the Supplementary Material, we provide a movie (movie1) presenting the full ensemble of experimental fluence cross sections where the differences between the regimes are very apparent.

For a pulse energy of 5 μJ, the main ring splits azimuthally into several peaks that rotate around the optical axis. This seems to correspond to the regime observed by another group in water^26^. The sense of rotation is determined by the sign of the vortex charge. A second movie (Supplementary Material, movie 2), based on experimental results, shows the high degree of symmetry of the rotation during nonlinear propagation at 5 μJ for m = 3 and m = −3. We note that in the linear case, no rotation was observed. Over 20 μJ, although one would expect multifilamentation, a novel regime appears, characterized by the fact that no continuous light channel is produced. Instead, multiple hot spots appear and disappear during the propagation with very limited rotation, resembling a speckle structure. Importantly, although very complex, this “speckle-like” regime is highly reproducible from shot to shot. Moreover, the same fluence distribution pattern was reproduced between identical experiments even when separated by several hours. This suggests that the speckle structure arises from inhomogeneity in the initial conditions rather than noise during propagation.

#### Spatial spectrum analysis

**Figure 5:**
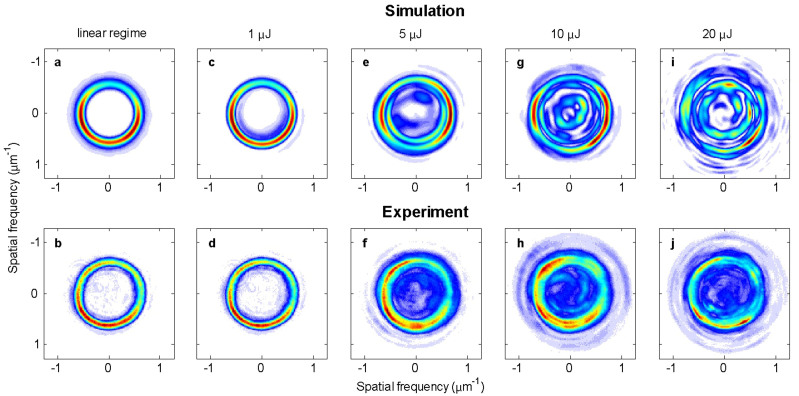
Spatial spectra of linear, stationary (1 μJ), rotating (5 μJ & 10 μJ), and speckle-like (20 μJ) regimes. We compare the far-field fluence distribution for numerical (top) and experimental (bottom row) results at propagation distance z = 1 mm. All parameters are identical to those used for figure 4.

Spatial spectrum provides an efficient tool to understand the propagation dynamics in the low conical angle case. Experimentally, the Fourier spectrum was measured at the focal plane of a lens inserted in the imaging path of the previous setup (see methods). In Fig. 5, we compare numerical and experimental Fourier-transforms of the beam shown in figure 4, at the propagation distance z = 1 mm. Numerical simulations use exactly the same input conditions as in figure 4. The first column (Fig. 5(a,b)) shows the linear regime where the Fourier transform of a Bessel vortex beam appears as ring with a radius *k_r_*_0_ = *k*sin*γ*.

Figures 5 (c,d) show the spatial spectrum for an input pulse energy of 1 μJ. A slight increase of the ring radius is characteristic of the propagation-invariant regime at high intensity, where, in the near-field, lobe compression occurs (see Fig. 2a). As the input pulse energy further increases, self-phase modulation and four-wave mixing modify the spatial spectrum and generate one or more rings with radii close to the initial one. For input pulse energies higher than 5 μJ, we observe the generation of spectral components with transverse wavevectors smaller than *k_r_*_0_ in a complex pattern. These waves are generated by nonlinear wave mixing. They were removed when Kerr index was numerically set to zero and they disappeared in experiments when the pulse duration was temporally stretched. These spectral components arise from a combination of four-wave mixing and self-phase modulation. Simulations showed that the nonlinear propagation radially modulates the beam resulting in the generation of several concentric rings in the far field, as for the case of zeroth order nonlinear Bessel beams^27^. When intensity perturbations are added to the input beam, modulation instability splits these rings azimuthally. This effect is more and more visible as the input pulse energy increases.

We note that in the rotating regime, between ~5 and ~20 μJ, the number of hot spots does not necessarily correspond to the vortex order: it evolves with propagation. The actual number of peaks depends on the growth rate of each azimuthal mode^10^.

At energies higher than 20 μJ, a number of azimuthally modulated rings are observed in the far field. They interfere in the near field and generate a highly complex, “speckle-like” pattern. The high degree of nonlinear cascading has already been observed to be deterministic^28^. Besides, it makes the propagation extremely sensitive to nonlinear coefficients and a relatively small quantitative discrepancy is therefore observed here between simulations and experimental results, while the qualitative behavior of speckle-like propagation is still present in both.

## Discussion

**Figure 6:**
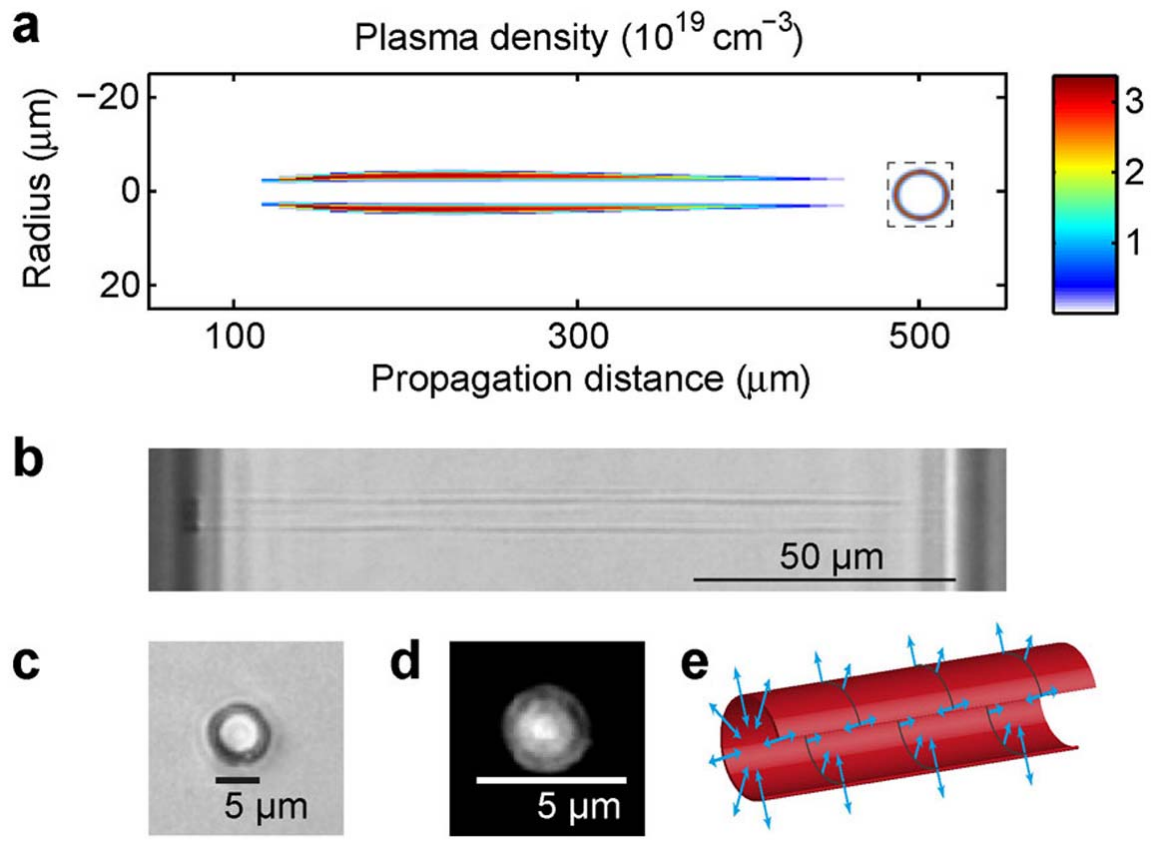
Applications to laser material processing. (a) Tubular plasma generated in the stationary regime. We show the longitudinal free-electron plasma density distribution, corresponding to the case of figure 3, at a pulse energy of 5 μJ. The inset shows the cross section of the plasma distribution at a distance of 300 μm (b) longitudinal view under transmission optical microscopy of a tubular damage produced in glass by single shot, high energy, stationary Bessel vortex, with m = 3, pulse energy 35 μJ and pulse duration 1 ps. The damage extends from one sample side to the other. (c) Transverse section of the damage observed in identical conditions the same beam at pulse energy of 20 μJ. The bright central region shows high index modification of the core of the tubular region. Remarkably, no light from the laser pulse has crossed this volume. (d) Image of near-field output guided light in the structure shown in (c), at an input wavelength of 632 nm. A ratio exceeding 20 dB is observed between the bright peak and the peripheral dark field outside. (e) Schematic view of the propagation direction of mechanical and thermal waves expanding outward and inward (arrows) from the excited tubular sheet volume (circle).

The propagation-invariant regime of Bessel Vortices opens numerous novel possibilities in the field of laser material processing. Indeed, we observed that this regime can be reached over a very wide range of parameters. Figure 6(a) shows a typical result of the longitudinal distribution of free-electron plasma in the propagation-invariant regime. Here, it corresponds to the case of the beam represented in figure 3(i) with a pulse energy of 5 μJ. The plasma distribution is longitudinally quasi-invariant over a range of 150 μm. The plasma cross section, shown in inset, peaks on the main intensity ring of the propagation-invariant Bessel vortex beam.

The tubular regime allows plasma generation on a very wide range of plasma densities and we note that density and geometrical parameters of the plasma tube can be controlled by six independent parameters (pulse energy, pulse duration, input Gaussian beam waist, cone angle, vortex order, laser central wavelength).

Figure 6(b) shows an image by optical transmission microscopy of the damage induced in 150 μm-thick glass slide (Corning 0211, see Methods section) by a single pulse with cone angle *γ* = 6.8° in samples, vortex order m = 3 and energy 35 μJ. The differences in refractive index and dispersion coefficient have a negligible impact on the propagation. Corning 0211 has a smaller bandgap (~4.2 eV) and the ionization avalanche threshold arises at lower intensity than in fused silica. The important difference between these materials is the thermal expansion coefficient (8.4 × 10^−6^ K^−1^ for Corning 0211 and 0.5 × 10^−6^ K^−1^ for fused silica).

The damage produced in glass is highly homogeneous with the shape of a cylinder extending from one side of the sample to the other. A pulse duration of 1 ps was chosen since the damage observed through optical transmission is much more visible than the one left by 100 fs, as already observed in previous work^16^. The damage produced in fused silica under identical conditions as those for Corning 0211 was qualitatively similar but the index modification is smaller. Figure 6 (c) shows the cross section of a similar damage, obtained at pulse energy of 20 μJ. The picture is obtained under transmission microscopy and it is apparent that the central core of the damage is brighter than the other part of the transparent sample, thus indicating that the refractive index has increased by material compression and melting. Importantly, no light from the laser pulse has illuminated this central core. This core actually guides visible light and the near field image is shown in Figure 6(d). From the numerical aperture of the coupled light, we estimate the refractive index change to be ~8 × 10^−4^. By using a similar approach as Ref. 23, we estimate the stopping distance of the shockwave to ~100 nm inside and outside the plasma tube^23^. A model of cylindrical heat diffusion shows that laser deposited energy on the plasma tube is enough to overcome the melting temperature over the tube diameter. We note that the much high thermal expansion coefficient of 0211 glass compared to fused silica generates a higher thermal stress. This explains the higher material modification observed in Corning 0211 glass.

Our approach provides means to write waveguides with a single shot in media where waveguide writing by Gaussian beams usually generates negative index modification. Hollow beams were previously used for this application in a regime with much smaller pulse energy, multishot illumination regime with continuous translation^29^. Here, an important novel step is that the high value of deposited energy in single shot allows the generation of material waves. Figure 6(e) shows schematically the propagation directions of inward and outward propagating *cylindrical material waves* generated from the tubular plasma. These waves are shockwaves, pressure waves and heat waves. The exact thermodynamical pathway yielding mechanical compression and material modification highly varies with radial energy distribution and material constants for heating, diffusion, thermal expansion and cooling after the plasma excitation. We found a variety of different damages depending on material and illumination parameters.

The tubular, propagation-invariant regime effectively de-couples light propagation from energy deposition within dielectric media. Our results are of primary importance because they open the way to control thermodynamics of materials under extreme conditions. Specifically, materials can be compressed and/or heated inside the hollow part of the beam, over arbitrary long distances, even if the material in the central core is of different composition as the dielectric where light propagates. In addition to very novel perspectives for laser material stress modification, high speed laser drilling and cutting of transparent materials, the multimegabar pressures generated by the explosion of dense plasmas from femtosecond pulses^30^ can be applied to the synthesis of novel material phases in non-negligible amounts.

In conclusion, we have shown numerically and experimentally the existence of tubular propagation-invariant waves at high power in dielectric solids based on femtosecond Bessel beams with topological vortex singularity. Accurate numerical simulations and novel experimental approach of beam imaging allowed us to exhibit the transition from propagation-invariant to rotating and “speckle-like” regimes by a combination of four wave mixing and modulation instability. We anticipate that the impact of our results will also be important for filamentation in gases and specifically for long-range filamentation and microwave guiding by filaments in atmosphere^31^. We expect that propagation-invariant conical vortex waves and the subsequent novel degree of control on plasma geometry will have a dramatic impact in several new fields in physics such as plasma guiding with plasma tubes, high-aspect ratio laser material processing, femtosecond laser waveguide writing and material compression.

## Methods

### Experimental setup

#### Bessel vortex beam

The experimental setup is based on shaping the beam of a 130 fs Ti:Sa laser with a spatial light modulator (SLM). The SLM plane is imaged through a telescope with a first lens L_1_ of focal length f_1_ = 1 m and a microscope objective (MO_1_, x20) placed in a 4f configuration. In the image of the SLM, light amplitude distribution is demagnified by a factor 110. As described in Ref. 32, the SLM phase mask applies a combination of the target phase and a reference phase so that the diffraction orders are spatially separated in the focal plane of lens L_1_. Spatial filtering allows us to filter out all undesired diffraction orders. The Bessel vortex beam onsets from the focal plane of the microscope objective.

#### Sample imaging and positioning

The samples (1 mm thick) were mounted on a 3 axis motorized XYZ stage (bidirectional repeatability 0.14 μm) and a two axis tilt clear-aperture piezo-mount. To image both the sample and the femtosecond beam, we placed a x50, NA 0.8 microscope objective (MO_2_) after the sample, on a separate translation stage (bidirectional repeatability 0.14 μm) to follow the sample z position along the optical axis. A CCD camera is placed in the focal plane of a f_3_ = 200 mm lens to image the sample with a magnification factor 55. Under white light illumination, the depth of field of sample imaging is less than 0.5 μm. The sample tilt compensation was then adjusted over the whole sample area (20 × 10 mm^2^) yielding a planarity better than 1 μm over 20 mm (50 μrad).

#### Fluence distribution imaging

For beam imaging, we use the same imaging system as for sample imaging. A high dynamical range (16 bits) CCD camera was electronically synchronized with the laser and imaged a single laser shot. Light intensity was attenuated by neutral densities placed before the camera and the camera gain was maintained to zero. Careful calibration of neutral densities, imaging setup transmission and camera response provides camera signal conversion in physical fluence (J/cm^2^).

Imaging a plane inside the sample would be an indirect measurement of the fluence distribution in this plane since amplitude and phase can be affected by nonlinear propagation after this plane. Our imaging procedure makes use of the fact that the nonlinear propagation regime in fused silica has a finite extent, smaller than the sample thickness. In addition, we limit ourselves to pulse energies where we measured the propagation in air was linear. We image the fluence distribution only at the sample exit side: we reconstruct the 3D fluence distribution by translating the beam within the sample and always image the fluence at the sample exit side.

The image of the SLM is first placed at the exit side of the sample, by translating the sample toward the laser source. The sample is illuminated by a single pulse and an image is recorded by our imaging system. Then, the sample is transversally shifted in plane by Δ*x* = 50 μm in order to illuminate a fresh part of the sample and moved along the optical axis by a distance Δ*z* = 2 μm for the high cone angle case and 5 μm for the low angle one. The imaging microscope objective MO_2_ is translated axially by the same amount to image the sample exit side. The fluence distribution measured at the exit side then corresponds to the one at a propagation distance nΔ*z* within the sample, where n is the sample index of refraction.

The spatial integration of fluence provides the pulse energy at each propagation distance, after correction of Fresnel losses. The ratio between the pulse energy before and after the high-intensity region provides the energy loss.

#### Far field imaging

Far field images were obtained by inserting a supplementary lens (f_2_ = 125 mm) between MO_2_ and L_3_ to perform an optical Fourier-transform.

#### Samples

We used high purity grade synthetic fused silica Lithosil® Q1 from Schott. The samples were 1 mm thick with a total thickness variation (TTV) less than 10 μm. For laser processing and results shown on figure 6, we used Corning 0211 glass, with thickness 150 μm.

### Modelling

#### Direct numerical simulations for beam propagation

The propagation model takes the canonical form of a unidirectional envelope propagation equation written in the spectral domain for the electric field envelope 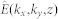:^22^


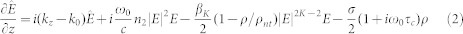
where 
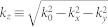
, *k*_0_ denotes the wavenumber in the medium of index *n*_0_ = 1.45 at central frequency *ω*_0_ = 2.4 × 10^3^ ps^−1^, *n*_2_ = 3.54 × 10^−16^ cm^2^/W the nonlinear coefficient for the optical Kerr effect, and *β_K_* = 6.8 × 10^−54^ cm^7^/W^4^ the cross section for multiphoton absorption of order *K* = 5.

In addition, the effects of high intensity are of interest for potential applications of conical vortex waves to laser energy deposition over a tubular structure. We therefore considered the generation of an electron-hole plasma, with density *ρ* described by the rate equation:


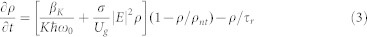
where *σ* denotes the cross section for inverse Bremsstrahlung from Drude model (electron effective mass coefficient is 0.64), *τ_c_* = 1.3 fs represents a phenomenological collision time, *U_g_* = 7.1 eV denotes the material bandgap, *ρ_nt_* = 2.1 × 10^22^ cm^−3^ is the initial neutral density of molecules and *τ_r_* = 150 fs the recombination time.

The last term in Eq. (2) represent plasma absorption and defocusing. Propagation distances are too short for dispersive effects to be relevant. Our beam propagation model therefore considers the electric field *E*(*x, y, z*) as time-independent and the electron density *ρ*(*x, y, z*) is determined as a function of intensity by solving Eq. (3) for a pulse with maximum intensity |*E*(*x, y, z*)|^2^ and fixed Gaussian pulse shape 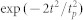 with *t_p_* = 110 fs. The input beam is a Gaussian beam (*r*_0_ = 35 μm) carrying a vortex charge *m* and a linear spatial phase equivalent to that induced by an axicon:


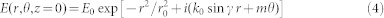


#### Eigenvalue problem for propagation-invariant conical vortex waves

From previous works on nonlinear Bessel beams^13^,^14^, we know that the key ingredients in the propagation equation for finding propagation-invariant states in the form of nonlinear Bessel beams are the nonlinear losses and the focusing/defocusing nonlinearity. We note that, for the present study, UPPE-based model provides the same results as Nonlinear Schrödinger equation (NLS) -based numerical model, which justifies the use of the NLS equation here. We rewrite our model in the form of a NLS equation suitable for the analysis of stationary solutions, i.e., with a diffraction operator expanded in cylindrical coordinates.


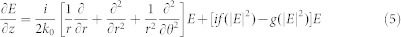
where *f*(|*E*|^2^) ≡ *k*_0_(*n*_2_/*n*_0_)|*E*|^2^ − *σ*/2*ω*_0_*τ_c_ρ* and *g*(|*E*|^2^) ≡ *β_K_*|*E*|^2*K*−2^/2 − *σρ*/2.

Without loss of generality, we looked for propagation-invariant solutions in the case *σ* ≅ 0, i.e., we considered only one physical effect of each type, namely, the optical Kerr effect and multiphoton absorption losses. We seek propagation-invariant beams carrying angular momentum, in the form *E*(*r, θ, z*) = *a*(*r*)exp[*i*(*ϕ*(*r*) + *mθ* − *δz*], with *δ* > 0 resulting in the set of ordinary differential equations for *a*(*r*) and *q*(*r*) ≡ *dϕ*/*dr*


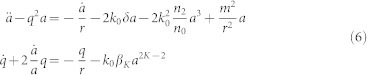
where dots means differentiation with respect to *r*. Equations (5) and (6), together with boundary conditions: *a*(*r*)→0 as *r*→0; *a*(*r*)→0 as *r*→+∞; *q*(*r*)→0 as *r*→0, represent an eigenvalue problem for the eigenvector *a*(*r*) and eigenvalue *δ*. In practice, a continuous spectrum of solutions exists for *δ* > 0, allowing us to equivalently fix the axial phase shift *δ* from the cone angle *γ*, through *δ* = (*k*_0_/2)sin^2^*γ*. We then solve numerically equations (5) and (6) with boundary conditions compatible with the behavior of a Bessel beam:


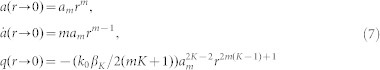
and we record the maximum intensity of the conical vortex wave for all solutions that decay back to zero as a Bessel beam. In this way, we map the region of existence of stationary conical Bessel waves in the plane (*I_peak_*, *γ*).

## Author Contributions

C.X. and V.J. equally contributed to this work. V.J., C.M. and A.C. developed the semi-analytical approach. V.J., C.M., A.C. and T.I. developed numerical codes and performed the numerical simulations. A.C. and F.C. developed the original idea and conducted the research along with T.I. and J.D. C.X., R.G., J.D. and F.C. developed the experimental setup. C.X. and I.O. acquired the experimental data that were processed by C.X. The manuscript was jointly written by all co-authors.

## Supplementary Material

Supplementary Information Supplementary information

Supplementary Information Movie 1. Comparison of the propagation regimes

Supplementary Information Movie 2. Comparison of propagation with positive and negative vortex charge

## References

[b1] Allen L. *et al.* Orbital angular momentum of light and the transformation of Laguerre-Gaussian laser modes. Phys. Rev. A 45, 8185–8189 (1992). 9906912 10.1103/physreva.45.8185

[b2] Grier D. G. A revolution in optical manipulation. Nature 424, 810–816 (2003). 12917694 10.1038/nature01935

[b3] Fürhapter S., Jesacher A., Bernet S. & Ritsch-Marte M. Spiral interferometry. Opt. Lett. 30, 1953–1955 (2005). 16092230 10.1364/ol.30.001953

[b4] Firth W. J. & Skryabin D. V. Optical solitons carrying orbital angular momentum. Phys. Rev. Lett. 79, 2450–2453 (1997).

[b5] Courtial J., Dholakia K., Allen L. & Padgett M. J. Second harmonic generation and the conservation of orbital angular momentum with high-order Laguerre-Gaussian modes. Phys. Rev. A 56, 4193–4196 (1997).

[b6] Soljačić M. & Segev M. Integer and fractional angular momentum borne on self-trapped necklace-ring beams. Phys. Rev. Lett. 86, 420–423 (2001). 11177845 10.1103/PhysRevLett.86.420

[b7] Mair A., Vaziri A. Weihs G. & Zeilinger A. Entanglement of the orbital angular momentum states of photons. Nature 412, 313–316 (2001). 11460157 10.1038/35085529

[b8] Allen L. Barnett S. M. & Padgett M. J. Optical Angular Momentum (Institute Of Physics Publishing., 2003).

[b9] Desyatnikov A. S., Torner L. & Kivshar Y. S. Optical Vortices and Vortex Solitons. Prog. Optics 47, 291–391 (2005).

[b10] Vuong L. T. *et al.* Collapse of optical vortices. Phys. Rev. Lett. 96, 133901 (2006). 16711987 10.1103/PhysRevLett.96.133901

[b11] Maleshkov G., Neshev D. N., Petrova E. & Dreischuh A. Filamentation and supercontinuum generation by singular beams in self-focusing nonlinear media. J. Opt. 13, 064015 (2011).

[b12] Polynkin P., Ament C. & Moloney J. V. Self-Focusing of Ultraintense Femtosecond Optical Vortices in Air. Phys. Rev. Lett. 111, 023901 (2013). 23889402 10.1103/PhysRevLett.111.023901

[b13] Porras M. A., Parola A., Faccio D., Dubietis A. & Di Trapani P. Nonlinear unbalanced Bessel beams: stationary conical waves supported by nonlinear losses. Phys. Rev. Lett. 93, 153902 (2004). 15524879 10.1103/PhysRevLett.93.153902

[b14] Polesana P., Franco M., Couairon A., Faccio D. & Di Trapani P. Filamentation in Kerr media from pulsed Bessel beams. Phys. Rev. A 77, 043814 (2008).

[b15] Bhuyan M. K. *et al.* High aspect ratio nanochannel machining using single shot femtosecond Bessel beams. Appl. Phys. Lett. 97, 081102 (2010).

[b16] Bhuyan M. K. *et al.* Single shot high aspect ratio bulk nanostructuring of fused silica using chirped controlled ultrafast laser Bessel beams. Appl. Phys. Lett. 104, 021107 (2014).

[b17] Paterson C. & Smith R. Higher-order Bessel waves produced by axicon-type computer-generated holograms. Opt. Commun. 124, 121–130 (1996).

[b18] Berry M. V. & McDonald K. T. Exact and geometrical optics energy trajectories in twisted beams. Journal of Optics A: Pure and Applied Optics 10, 035005 (2008).

[b19] Jarnac A. *et al.* Whole life cycle of femtosecond ultraviolet filaments in water. Phys. Rev. A 89, 033809 (2014).

[b20] Jukna V. *et al.* Filamentation with nonlinear Bessel vortices. Opt. Express 22, 25410–25425 (2014). 25401574 10.1364/OE.22.025410

[b21] Porras M. A. & Ruiz-Jiménez C. Non-diffracting and non-attenuating vortex light beams in media with nonlinear absorption of orbital angular momentum. J. Opt. Soc. Am. B 31, 2657–2664 (2014).

[b22] Couairon A. *et al.* Practitioner's guide to laser pulse propagation models and simulation. Eur. Phys. J. Spec. Top. 199, 5–76 (2011).

[b23] Gamaly E. G. The physics of ultra-short laser interaction with solids at non-relativistic intensities. Phys. Rep. 508, 91–243 (2011).

[b24] Polesana P. *et al.* Observation of conical waves in focusing, dispersive, and dissipative Kerr Media. Phys. Rev. Lett. 99, 223902 (2007). 18233286 10.1103/PhysRevLett.99.223902

[b25] Faccio D. *et al.* Nonlinear light-matter interaction with femtosecond high-angle Bessel beams. Phys. Rev. A 85, 033829 (2012).

[b26] Shiffler S., Polynkin P. & Moloney J. Self-focusing of femtosecond diffraction-resistant vortex beams in water. Opt. Lett. 36, 3834–3836 (2011). 21964113 10.1364/OL.36.003834

[b27] Gadonas R. *et al.* Self-action of Bessel beam in nonlinear medium. Opt. Commun. 196, 309–316 (2001).

[b28] Majus D., Jukna V., Valiulis G. & Dubietis A. Generation of periodic filament arrays by self-focusing of highly elliptical ultrashort pulsed laser beams. Phys. Rev. A 79, 033843 (2009).

[b29] Long X., Zhao W., Stoian R., Hui R. & Cheng G. Writing of stressed waveguides with tubular depressed cladding using femtosecond hollow beams. Opt. Lett. 37, 3138 (2012). 22859111 10.1364/OL.37.003138

[b30] Juodkazis S. *et al.* Laser-induced microexplosion confined in the bulk of a sapphire crystal: evidence of multimegabar pressures. Phys. Rev. Lett. 96, 166101 (2006). 16712248 10.1103/PhysRevLett.96.166101

[b31] Châteauneuf M., Payeur S., Dubois J. & Kieffer J.-C. Microwave guiding in air by a cylindrical filament array waveguide. Appl. Phys. Lett. 92, 091104 (2008).

[b32] Froehly L., Jacquot M., Lacourt P.-A., Dudley J. M. & Courvoisier F. Spatiotemporal structure of femtosecond Bessel beams from spatial light modulators. J. Opt. Soc. Am. A 31, 790–793 (2014). 10.1364/JOSAA.31.000790 24695141

