# Supplementary material for: Tubular filamentation for laser material processing
Source: Sci Rep. 2015 Mar 10;5:8914. doi: 10.1038/srep08914 (PMC4353995; doi:10.1038/srep08914)
Supplement: Supplementary Information [file srep08914-s1.pdf]

Tubular filamentation for laser material processing

Chen Xie[1], Vytautas Jukna[2,3], Carles Milián[2], Remo Giust[1], Ismail Ouadghiri[1], Tatiana Itina[3], John M. Dudley[1], Arnaud Couairon[2] and Francois Courvoisier[1,*]

(1) Département d'Optique P. M. Duffieux, Institut FEMTO-ST, UMR 6174 CNRS Université de Franche-Comté, F-25030 Besançon cedex, France

(2) Centre de Physique Théorique, CNRS, Ecole Polytechnique, F-91128 Palaiseau, France

(3) Laboratoire Hubert Curien, UMR CNRS 5516, Université de Lyon, Université Jean Monnet, F-42000 Saint-Etienne, France

francois.courvoisier@femto-st.fr

Supplementary Information

Video Legends:

Movie 1: Experimental propagation in linear, propagation invariant, rotating and "speckle-like" regimes. This data is the same as the one shown in figure 4.

Movie 2 : Comparison of the linear and rotating regimes for a vortex charge m=3 and m=-3, for pulse energy 5μJ.
